# A Noisome Stench

**Published:** 1900-10

**Authors:** 


### A NOISOME STENCH.

THE City of Buffalo has been the sufferer of late from an abuse of privilege that has brought distress to its people and shame to those responsible for it or who caused it. About the middle of August one of the large grain elevators that skirt the harbor—the Dakota—was destroyed by fire, and about 600,000 bushels of wheat and corn were scorched or wet. It became necessary in order to dry this grain to distribute it among the malt houses where facilities were ample for the purpose. The odor that has been given off by this sour grain is indescribable. It is sickening in the extreme; it nauseates the well and prostrates the sick. It disturbs the appetite and destroys sleep. It is a menace to good temper and a disgrace to the civilisation of the period. It has smirched the good name of our fair city and brought dishonor to its municipal administration.

If we were asked to describe the odor that has so completely disrupted the ordinary course of affairs and played such havoc with the amiability of our citizens, we should be compelled to say that it resembled the effluvium of night soil, the stench of rendering works and the tainted atmosphere of the tannery—all combined and each intensified about ten-fold. To live in such an aggregation of stinks for a whole month is enough to send one to a mad house with curses on his lips for the mercenary men who are responsible for this outrage upon common decency.

Visitors to our city have been driven hurriedly away and inhabitants have sought relief in nearby summer resorts, crying in their exodus, Oh! Lord, how long?

That we do not exaggerate the importance of this subject or its effects on our people we quote the following letter addressed to the mayor by one of our most prominent and influential women:

"*To the Mayor of the City of Buffalo:*

"Sir—Three weeks ago an overpowering stench so polluted the atmosphere, that I had recourse at once to the police to learn its cause, and then to the Health Commissioner, to the Corporation Counsel, and to various city officials to ask its abatement.

"The people have complained of it, the papers have denounced it, but we are still not rid of this abomination.

"No portion of our beautiful city is free from its permeating influence, and from the park system to the burned elevators our citizens are suffering, not only mentally but physically, from the foul effects of this decayed vegetable matter, which some are pleased to call 'drying grain!'

"Those in the immediate vicinity of the elevators are the most constant sufferers, and for these, as well as those throughout our city who seem to be unable to speak for themselves, I appeal to you as head of the municipal government to take such steps as shall immediately stop the present outrageous course of proceedings, and put an end to not only a senseless, but to a criminal bickering, which subjects us all to an unwholesome and sickening atmosphere, while a few dollars more or less are being made by a few men at the expense of our city, and its citizens.

"This appeal I make to you as Mayor of the City of Buffalo, and if the names of those for whom I speak will give added weight, I can send you hundreds of those who will be glad to sign them.

"Very respectfully yours,

"MARIA M. LOVE."

"September 6, 1900."

We cannot understand how this deplorable state of things was allowed to proceed from day to day in spite of the protests that were made by newspapers and individuals. It ought not to be possible for such a state of affairs to arise in a well-governed city, and if the advice of Health Commissioner Wende had been heeded a few years ago, we should not have been subjected to such distress, humiliation and disgrace. It will, no doubt, cost the Pan-American Exposition thousands of admissions next year, for we have heard many persons say that it would prevent their attendance and likewise the coming of as many friends as they could influence. It is not easy for a city to rid itself of such a reputation as Buffalo obtained during the midsummer of 1900. It will prevent people of leisure and means from coming here to reside, or to spend a few weeks in recreation, and in various other ways will retard our progress. It is a piece of provincialism that ought to teach a lesson, even if it is expensive.

The only wonder is that the Mayor did not rise in his might, with all the power at his command, and stamp out the evil when it first appeared. In another place the nuisance is dealt with from another viewpoint.

